# Validation of POSSUM, P-POSSUM and the surgical risk scale in major general surgical operations in Harare: A prospective observational study

**DOI:** 10.1016/j.amsu.2019.03.007

**Published:** 2019-03-27

**Authors:** Allan Ngulube, Godfrey I. Muguti, Edwin G. Muguti

**Affiliations:** Department of Surgery, College of Health Sciences, University of Zimbabwe, Box A167, Avondale, Harare, Zimbabwe

**Keywords:** Audit, POSSUM, Risk scoring system, POSSUM, Physiological and Operative Severity Scoring for the enUmeration of Mortality and Morbidity, P-POSSUM, Portsmouth-POSSUM, SRS, Surgical Risk Scale, ASA, American Society of Anesthesiologists

## Abstract

**Background:**

Raw mortality and morbidity, though commonly studied in surgical audit can nonetheless be misleading because of differences in preoperative and intraoperative findings of patients. There are some common scoring systems specifically designed to cater for case mix but these have not been tried locally. This study sought to validate these scoring systems and hopefully adopt them for our teaching hospitals.

**Materials and methods:**

A prospective observational cohort study was conducted at two central hospitals in Harare Two hundred and two patients undergoing a variety of major general surgical operations were recruited into the study. Results of physiological and intraoperative parameters collected from the patients’ records were scored according to POSSUM, P-POSSUM and SRS scores. Predicted mortality and morbidity rates of all these subjects were then compared to the observed rates.

**Results:**

One hundred and eighty one patients participated (123 males, 58 females). Using the POSSUM morbidity score, the observed versus expected (O: E) ratio of 0.88 showed no difference (p = 0.970). Using POSSUM, P-POSSUM and SRS mortality scores, O: E ratios of 0.74, 1.06 and 1.0 respectively were obtained, the differences were not significant (p = 0.650, p = 0.987 and 0.730). All three scores were comparable on the Receiver Operating Characteristic curve. The Physiological score independently predicted mortality (p < 0.00001).

**Conclusion:**

POSSUM, P-POSSUM and SRS scores are comparable and suitable for estimating outcomes after major surgery in Harare. A larger study inclusive of low risk patients is needed to generalise these findings across Zimbabwean patients.

## Highlights

• POSSUM accurately predicts morbidity.
• POSSUM, P-POSSUM and SRS accurately predict mortality in Harare.
• POSSUM, P-POSSUM and SRS are comparable for predicting outcomes in Harare.
• Mortality is significantly associated with preoperative physiological score.

## 1 Introduction

Surgical audit based on mortality and morbidity, has long been known not only as a research generating tool but also as a crude method to assess a surgical unit's performance and in parts of the developed world today, it is compulsory [1]. Although good surgical technique is paramount in reducing adverse outcomes, the ultimate outcome is also dependent on the physiological state of the patient, the operative severity and peri-operative support services [2]. These critical factors make it difficult to assess a unit's performance based only on the raw outcome figures, therefore an objective method that also takes into account case mix is needed.

With these concerns in mind, Copeland et al. (1991) developed the **P**hysiological and **O**perative **S**everity **S**coring for the en**U**meration of **M**ortality and Morbidity (POSSUM) risk-adjusted scoring system as a method of normalizing data so that direct comparison of patient outcome can be made despite differences in case-mix. POSSUM score calculation is based on the use of 12 physiological and 6 operative variables from the patient, which are graded as 1,2,4 or 8 based on their magnitude then summated to form a physiological score (PS) and operative severity score (OSS)(Table 1). The PS and OSS are then factored into predictor equations which predict the risk of mortality and morbidity [3]. (equations (1), (2), (3)) The original POSSUM surgical scoring system was found to consistently overestimate the mortality rate in low risk patients [4] thus a modification, the Portsmouth-POSSUM (P-POSSUM), was made which claimed to produce a closer fit with the observed outcomes [5]. Another separate team subsequently developed the Surgical Risk Scale (SRS) which it claimed to be better as it requires less data all obtainable preoperatively and also fits better in predicting death for very low risk patients (Table 1) [6]. The SRS uses 3 parameters that are also graded by magnitude and summated to form a surgical risk score which is then factored into a SRS mortality predictor equation(equation (4)).

**Table 1:** Variables used to calculate the POSSUM and SRS risk scores.

POSSUM Score variables		Surgical risk score		
Physiological score	Operative severity score	*ASA Grade	BUPA	NCEPOD
Age	Type of Operation	1	Minor 1	Elective 1
Cardiac Status	Number of Procedures	2	Intermediate 2	Scheduled 2
Respiratory Status	Operative Blood Loss	3	Major minus 3	Urgent 3
ECG Status	Peritoneal Soiling	4	Major Plus 4	Emergency 4
Systolic Blood Pressure	Malignancy Status	5	Complex Major 5	
Pulse	Timing of Operation			
Haemoglobin				
*ASA – American Society of Anaesthesiologists				
BUPA – British United Provident Association				
NCEPOD - National Confidential Enquiry into Patient Outcome & Death				
White Cell Count				
Urea				
Sodium				
Potasium				
Glasgow Coma Scale				

The POSSUM and SRS risk prediction models have been tested and validated in various centres in the developed world and in some developing countries but there are no locally recorded studies to evaluate use of these formulae in the Zimbabwean patients despite their obvious advantages. This study sought to validate POSSUM, P-POSSUM and also the SRS and thus adopt their use in surgical audit in Zimbabwe's teaching hospitals.

The specific objectives were

1. To determine if there is any significant difference between observed versus predicted operative mortality and morbidity scores in Harare using POSSUM, P-POSSUM and SRS.
2. To determine which perioperative risk factors have the greatest impact on mortality and morbidity.

## 2 Materials and methods

A prospective observational cohort study was done with a minimum sample size of 166 using the Dobson formula. The study was conducted at Parirenyatwa Group of Hospitals (PGH) and Harare Central Hospital (HCH) over a 9 month period from January to September of 2015. The study included all consecutively admitted patients aged 18 years and above undergoing at least a major general surgical procedure as defined by the British United Provident Association [7], with timing ranging from elective to emergency. Patients were excluded if below the age of 18 years, if managed conservatively, if it was a day case or any procedure categorized as minor and any case falling outside the scope of general surgery. Those also excluded were patients with more than 1 missing result or those requiring admission into a critical care unit post operatively but failed because of shortage of beds and those operated by surgical trainees with less than 2 years experience.

Using a predesigned data collection tool, results from investigations done immediately preoperatively plus operative findings and post operative histology were collected. Complications, as defined by Copeland et al. [3], were recorded as observed by the attending surgeons with confirmatory tests where necessary. Patients were followed up for a month in Outpatients Department (OPD) and a follow up phone call was done for those not available for review. The actual calculation for the risk scores was done with a computer program utilising the stated formulae (Equations (1), (2), (3), (4))). The calculated risk scores for individual patients were stratified according to magnitude then compared with the actual observed number of mortalities or morbidities in each category with Chi-Square as a test for significance at 95% significance**.** Regression analysis of risk factors contributing to mortality and morbidity was also done with appropriate calculations for significance testing using statistical software.

Equation (1): POSSUM equation for morbidity:

(1) $$\ln\frac{R}{1-R}=\;-5.96+\left(0.16\times physiological\;score\right)+\;\left(0.19\;operative\;severity\;score\right)$$

Equation (2): POSSUM equation for mortality:

(2) $$\ln\frac{R}{1-R}=\;-7.04+\left(0.13\times physiological\;score\right)+\;\left(0.16\;operative\;severity\;score\right)$$

Equation (3): P-POSSUM equation for mortality:

(3) $$\ln\frac{R}{1-R}=-9.065+\left(0.1692\times physiological\;score\right)+\left(0.155\;operative\;severity\;score\right)$$

Equation (4): Surgical risk scale equation for mortality:

(4) $$\ln\frac{R}{1-R}=-9.81+\left(0.84\times SRS\;score\right)$$

(R is mortality or morbidity risk) [6,8].

## 3 Results

**Fig. 1:**
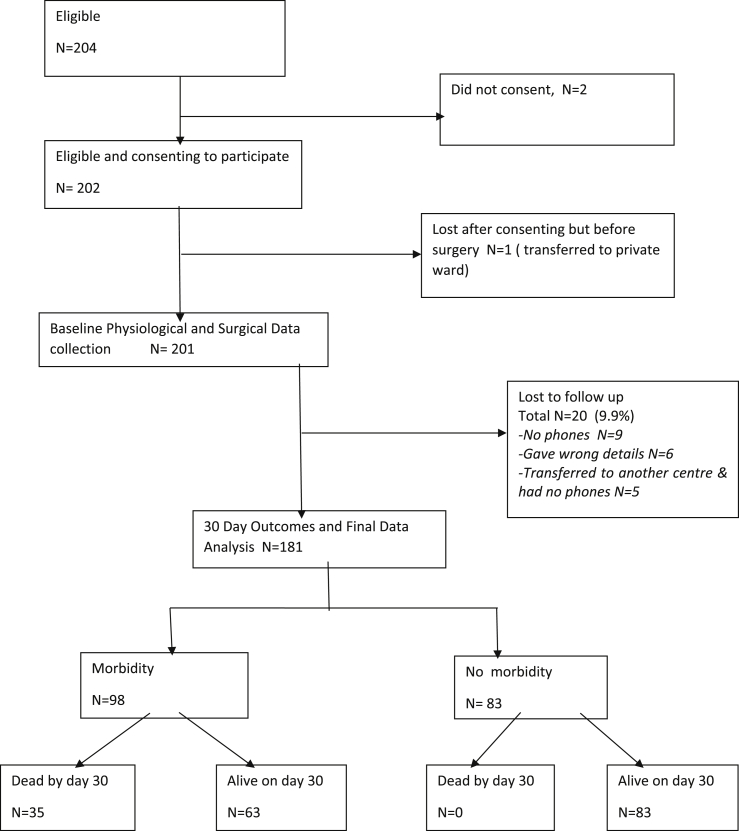
Flow chart.

The recruitment of patients is demonstrated in Fig. 1.

**Fig. 2:**
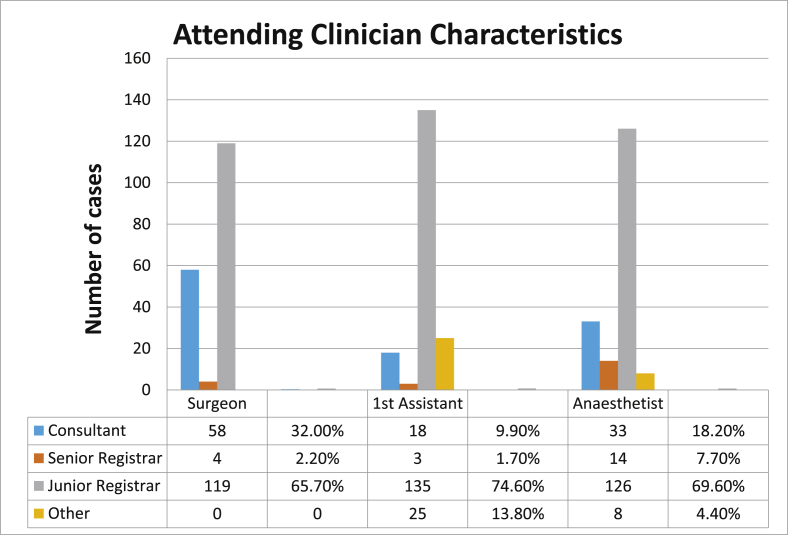
Attending clinician's characteristics.

The 181 study participants were 123 males (68%) and 58 females (32%). The age ranged from 18 years to 87 years with mean age of 48 (SD 17.7). The mean age for males was 47 (SD 18.7) and the mean age for females 50 (SD 15.5). The top 4 indications for surgery were peritonitis from appendiceal rupture or visceral perforation (26%), Sigmoid Volvulus (11%), Colorectal tumours (8.8%) and Small Bowel Obstruction (8.3%). The attending clinicians are illustrated in Fig. 2.

**Fig. 3:**
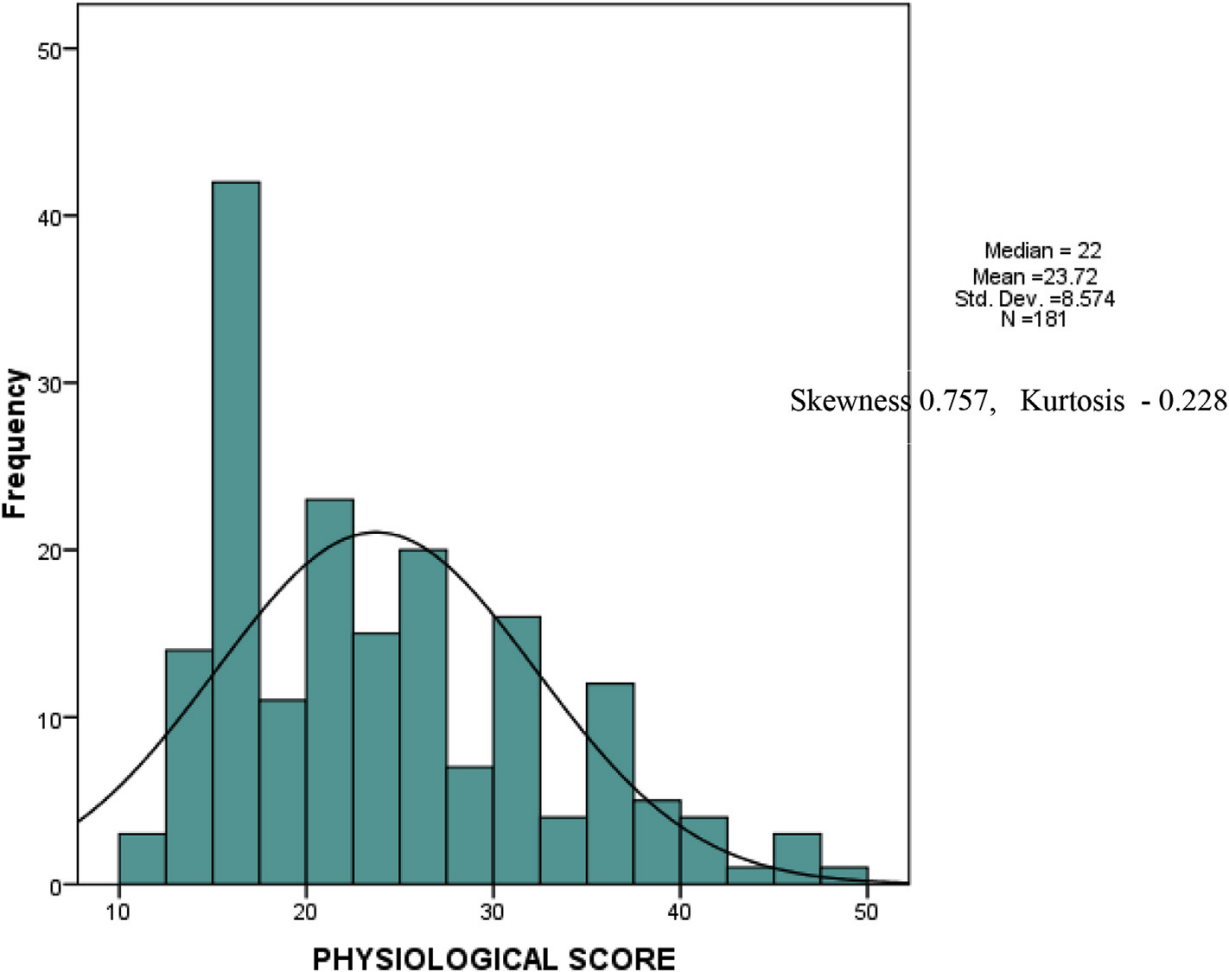
Distribution of preoperative physiological score.

A preoperative ASA score of 3 or more was obtained in 112(61.9%) patients and it correlated with mortality (p < 0.0001). The physiological score distribution is shown in Fig. 3. The median operative severity score (see equations (1), (2), (3))) was 15 (Q1 = 13, Q3 = 20). Thirty five of 181 (19.3%) (17 PGH, 18 HCH) patients had died of surgery related problems after a 30 day follow up. The proportion of cases and the mortalities in each CEPOD class are shown in Table 2.

**Table 2:** Mortality within CEPOD class.

	Elective		Scheduled		Urgent		Emergency
	n	(%)	n	( %)	n	(%)	n
Alive	28	(96.6)	39	( 83.0)	79	(76.0)	0
Died	1	( 3.4)	8	(17.0)	25	(24.0)	1
Total	29	(100)	47	(100)	104	( 100)	1
% of All cases	16		57.5		26		0.5

The overall morbidity was 54% and was noted to increase from 20.7% to 66.3% from elective to emergency surgery respectively. The most frequent complications were septic shock and superficial surgical site infection at 24.6% each followed by renal failure at 13.1% of all complications. Comparison of observed and expected POSSUM morbidity rates was done and an observed to expected ratio (O: E) of 0.88 was obtained and there was no difference (χ [2] = 1.52, 9d, P = 0.970). The area under the curve (AUC) for POSSUM morbidity score was 0.775 (p < 0.0001). Evaluation of POSSUM mortality rates yielded an O: E of 0.74 (χ [2] = 6.878, 9 d, p = 0.650). P-POSSUM mortality rates analysis is represented in Table 3 below and it yielded an O: E ratio of 1.06 (χ [2] = 2.25, 9 d, P = 0.987).

**Table 3:** Observed versus expected rates using P-POSSUM mortality score.

Mortality risk %	Number of Patients	Observed number of deaths	Expected Number of deaths	O:E
≤10	118	8	6	1.33
>10 to ≤20	15	3	2	1.50
>20 to ≤ 30	7	3	2	1.50
>30 to ≤40	11	3	4	0.75
>40 to ≤ 50	11	5	5	1.00
>50 to ≤60	5	3	3	1.00
>60 to ≤70	7	5	5	1.00
>70 to ≤80	4	2	3	0.67
>80 to ≤90	2	2	2	1.00
>90 to ≤100	1	1	1	1.00
Total	181	35	33	1.06

An O/E ratio of 1.00 indicates outcomes as expected and less than 1.00 indicates outcomes better than expected and >1.00 outcomes worse than expected.

Evaluation of SRS Mortality rates gave an O:E ratio of 1.00 and the difference was not significant (χ [2] = 0.119, 1d, p = 0.730.).

**Fig. 4:**
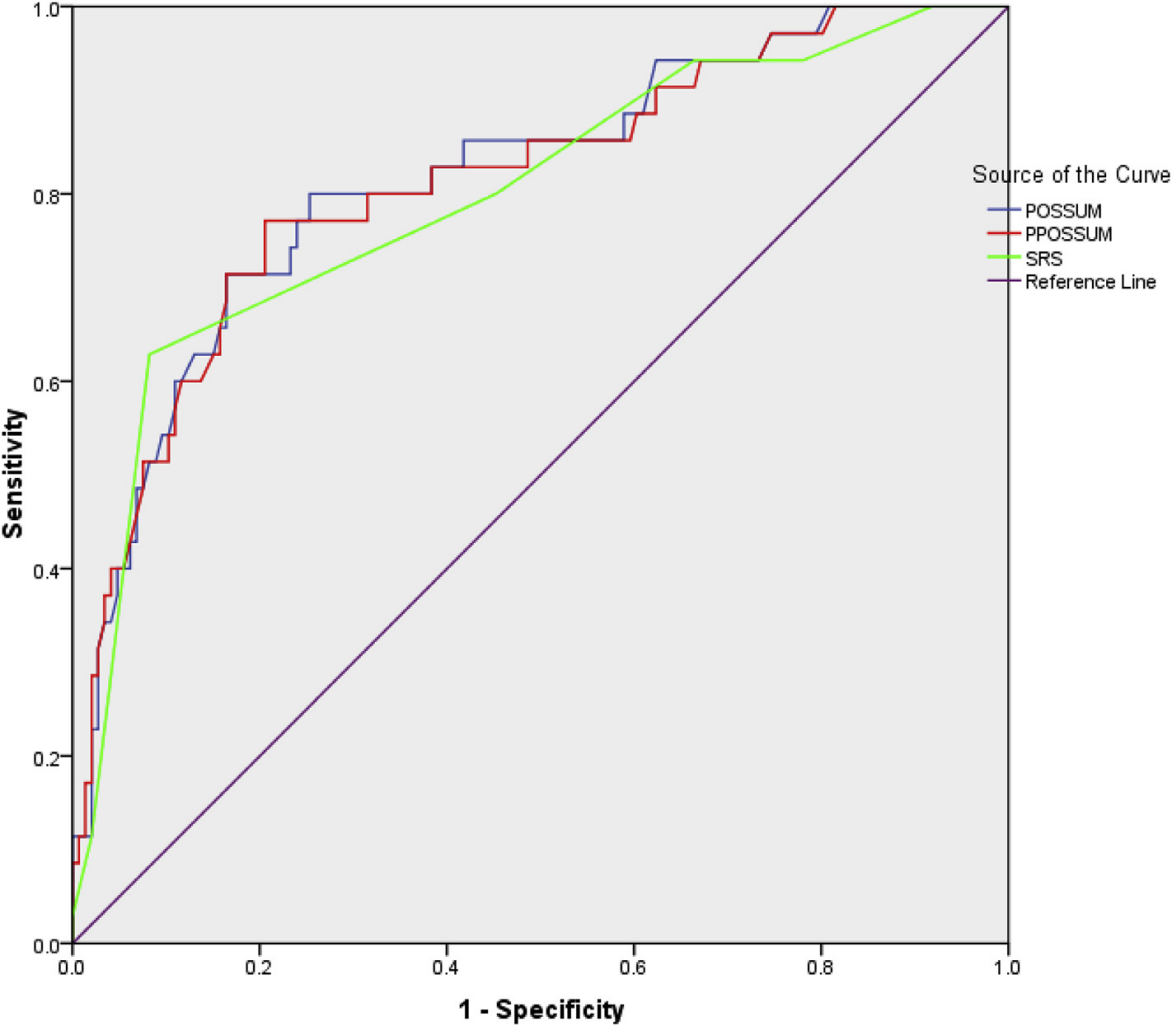
Receiving Operating Characteristic Curve (ROC) for POSSUM, P-POSSUM and SRS scores.

The receiving operating characteristic curve comparing all three scores is indicated in Fig. 4 and there was no difference in the 3 scores area under curve (AUC - POSSUM 0.818, SRS 0.799, P-POSSUM 0.814 p < 0.000).

Multivariate logistic regression for factors contributing to actual mortality is demonstrated in Table 4. On univariate analysis of the individual composite variables of the POSSUM and SRS scores, we found that the ASA score, the physiological score and operative severity scores were correlated significantly with mortality with (p < 0.00001), p < 0.00001 and 0.0036 respectively. Univariate regression analysis of confounding factors showed HIV status (p = 0.829), Diabetes Mellitus (p = 0.386), attending surgeon (p = 0.872) and attending hospital (p = 0.460).

**Table 4:** Multivariate regression analysis for mortality.

Variable	Odds Ratio	95%	C.I.	Coefficient	S.E.	Z-Statistic	P-Value
Age	0.562	0.2391	1.321	−0.5762	0.436	−1.3214	0.1864
Blood Loss	1.1099	0.8058	1.5289	0.1043	0.1634	0.6383	0.5233
Blood Pressure	1.0587	0.772	1.452	0.0571	0.1612	0.354	0.7233
Cardiac	1.8307	1.1202	2.9916	0.6047	0.2506	2.413	0.0158
Electrocardiogram	1.6415	1.0556	2.5528	0.4956	0.2253	2.2	0.0278
Glasgow Coma Scale	2.1264	0.6527	6.9277	0.7544	0.6026	1.2519	0.2106
Haemoglobin	1.1471	0.9574	1.3745	0.1373	0.0922	1.4883	0.1367
Potassium	1.0021	0.6717	1.4949	0.0021	0.2041	0.0103	0.9918
Malignancy Status	1.3202	1.0112	1.7235	0.2777	0.136	2.0419	0.0412
Timing of operation	1.2863	0.7833	2.1123	0.2518	0.2531	0.9949	0.3198
Multiple Operations	0.9755	0.5981	1.5911	−0.0248	0.2496	−0.0994	0.9208
Sodium	0.8513	0.4911	1.4758	−0.1609	0.2807	−0.5734	0.5664
Operation Severity	12.1773	2.1594	68.670	2.4996	0.8825	2.8323	0.0046
Pulse	1.4071	1.0774	1.8375	0.3415	0.1362	2.5076	0.0122
Respiratory	1.0418	0.8318	1.3048	0.0409	0.1149	0.3565	0.7215
Peritoneal Soiling	1.0524	0.8506	1.3021	0.051	0.1086	0.4699	0.6384
Urea	1.1774	0.9316	1.4879	0.1633	0.1194	1.3671	0.1716
White Cell Count	0.7408	0.4145	1.3239	−0.3	0.2962	−1.0128	0.3112

After performing a multivariate logistic regression for factors contributing to actual morbidity, the risk factors that have the greatest impact on morbidity were malignancy (p = 0.0356) and mode or when the operation was done (elective vs emergency) (p = 0.0131) with 95% confidence. Gross peritoneal soiling was identified as a risk factor for morbidity after univariate analysis, p = 0.0174. On univariate analysis, the ASA score POSSUM Physiological and Operative scores were correlated significantly with morbidity with p-values <0.0000, 0.002 and 0.007 respectively while the possible confounders, HIV status (p = 0.677), Diabetes Mellitus (p = 0.969), attending surgeon (p = 0.627) and attending hospital (p = 0.742) were not significant enough to affect the outcome.

## 4 Discussion

King Hammurabi of Ancient Babylon decreed the cutting off of the hands of ‘poorly' performing surgeons, a practice which cannot be justified today because auditing surgical performance based on mortality rates without risk adjustment for patient factors is grossly misleading [8]. A perfect risk adjusting scoring system does not exist, but the scores used in this study are easy, reproducible and we believe are applicable in the Zimbabwean patient.

In our study, we assessed the validity of POSSM, P-POSSUM and SRS in 201 major general surgical procedures, with a 10% loss to follow up leaving 181 patients for final analysis. In keeping with some centres in Africa most surgery is done on males showing a 2:1 male to female ratio [[9], [10], [11]]. Our mean age was 48 years (SD 17.7) which was considerably higher than other studies on POSSUM scoring in African patients e.g. Kitara et al. in Uganda and Mohammed in Sudan who had mean ages of 40 years and 28 years respectively [12,13].

Using the CEPOD classification 57% were urgent cases, 26% scheduled and 16% were elective cases, if reclassified by POSSUM score, this would translate to 65% of patients operated as an emergency and 35% as elective. This seems to be the trend in low resource settings where most of the major surgical work is in dealing with emergencies [12].

Most of the surgery in our study, 65.7%, was done by trainee surgeons in the absence of a consultant, this mirrors the results of Kitara in Uganda [12], but contrasts with Mohammed's study in Khartoum, Sudan where consultants, senior registrars and junior registrar each operate on 15.9%, 50.4% and 33.6% of all patients respectively [13]. Importantly from our study we find that the level and experience of clinicians did not seem to have an impact on mortality and morbidity (p = 0.872). We therefore postulate that surgical trainees may possibly use these scoring systems for longitudinal assessment of their own performance.

It has also been debated in literature that differences in individual surgeon versus the surgical team or hospital are risk factors in mortality [2]. In our study even though HCH had proportionally more mortalities (18 of 83 procedures) than PGH (17 of 98), the difference was not significant (p = 0.460). The use of multiple surgeons however meant we did not have sufficient numbers to compare between individual surgeons. However, it must be stated that even if there were enough numbers to do this comparison, one of the inherent weaknesses of POSSUM, adjusting for case mix, prevents such kind of analysis [6].

From our study and also in keeping with other studies in Africa, the most common indication for operation is peritonitis [9]. Not surprisingly infectious complications were the most frequent observed morbidity. Notably, on further analysis, septic shock requiring inotropic support was also the most commonly observed complication in patients that eventually died.

The operative scores in our study ranged from 9 to 37 with a median of 15 (Q1 = 13, Q3 = 20).Our physiological score was positively skewed with a median score of 22 (Q1 = 16, Q3 = 30). Our mean physiological score was similar to that in Sudan [13] though ours had a wider range. This indicates that many of our patients presented late and with severely deranged physiology. As can be expected, our morbidity was noted to increase from 20.7% to 66.3% from elective to emergency surgery respectively. Our overall morbidity of 54%, though similar to that found in Uganda [12], seems high just by looking at raw figures however our centres did slightly better than predicted by the score (O: E 0.88) with no statistically significant difference between observed and expected morbidity (χ [2] = 1.52, 9d, P = 0.970). This supports the need for a scoring system since one may conclude that the complication rate is too high without actually looking at the case mix. The AUC for POSSUM morbidity score is 0.775 (p < 0.0001) agreeing with existing literature that shows that the score has good discrimination for picking those who will get a morbidity [14].

In our study, risk factors with the greatest impact on morbidity are malignancy (p = 0.0356) and timing of the operation (i.e. elective vs emergency) (p = 0.0131). Similar factors have been identified in other studies and a suggestion has been made to the effect that correction of these factors preoperatively greatly changes the outcome [9]. Univariate regression analysis of possible confounding factors like HIV status (p = 0.677), Diabetes Mellitus (p = 0.969), attending surgeon (p = 0.627) were not significant enough to affect the outcome. Concerning HIV status we agree with findings by Cakala et al. that HIV status does not influence surgical outcomes of admitted patients [15]. It must however be noted that, only 108 of our 181 (59.7%) patients had a known HIV status and those with confirmed HIV infection had an unknown immunological status and viral loads. Also those with diabetes consisted 5.5% of the study population however their long term glycaemic control was unknown.It would be interesting to research the effect of increased viral load or poor glycemic control on POSSUM score interpretation.

Both the physiological score and operative severity scores correlated significantly with morbidity with p-values 0.002 and 0.007 and also with mortality with p-values <0.00001 and 0.0036 respectively. Of importance is that, this supports the observation from other papers suggesting that the physiological score taken in isolation can be used preoperatively to risk stratify the patients with good sensitivities [16,17]. We therefore believe that the physiological POSSUM score can be used for preoperative counseling of patients and allocation of resources in resource constrained areas.

The ASA score has been questioned because of its subjectivity and also because of its inability to predict mortality for individual to individual basis [14,18]. In our study, 61.9% of our patients had a preoperative ASA score of 3 or more of and it significantly predicted mortality p < 0.00001. This agrees with the study by Chu et al. in low resource settings where ASA score greater than 3 correlates with mortality [19]. We therefore agree with the usefulness of the ASA score in stratifying the risk of major surgery at our centres.

Our overall mortality of 19.3% (35/181) though similar to the results of Vallabha [20] and Tekkis [21] seems to be higher than quoted in other studies on major surgery [9]. However our mortality rate needs to be interpreted in the context of being only for major surgery and that this study was skewed towards emergencies, some with quite advanced disease. When our mortality is distributed according to CEPOD class (Table 2), of the 29 patients operated purely as elective cases, only 1 patient died giving an elective case mortality rate of 3.5%, which could be comparable with other literature [2], however the numbers may be too small to draw conclusions.

Mulitivariate logistic regression of factors significantly contributing to actual mortality identified cardiac status (p = 0.0158), ECG (p = 0.0278), malignancy stage (p = 0.0412), pulse (p = 0.0122) and operative severity (p = 0.0046) (Table 4). Some of these factors were also identified as being major contributors to mortality by Raut et al. in their study [9].

In our study, using similar statistical analysis methods for both POSSUM and P-POSSUM, the P-POSSUM mortality score was closer fitted to predict mortality d (O: E = 1.06, χ [2] = 2.25, P = 0.987) than POSSUM mortality score which somewhat overpredicted the mortality with O:E ratios of 0.74, the difference however was not statistically significant (χ [2] = 6.878, 9 d, p = 0.650). Other literature exists that suggests that using the same method of analysis for POSSUM and P-POSSUM does not give the same closeness of fit even though in our study POSSUM still fitted [22].

The area under the ROC curves for POSSUM, SRS and P-POSSUM showed no statistically significant difference and all were close to 80% of the area showing that all the three scores have good discrimination for picking those who will become a mortality [14]. Overall, the results for all three scores did not show any difference between observed versus expected outcome. Cochrane's rules of interpreting χ [2] require that there be a minimum of five predicted events in 80% of risk ranges of strata [23], which wasn't the case all the time in our study because of the relatively low numbers. Therefore in concurrence with what was noted by Nichols et al. in their study [24], one would be careful to generalise these findings to everyone undergoing surgery at our centres. If funding is available a much larger study including the lower risk stratas would need to be done.

### 4.1 Limitations

i. The study had no funding and as a result some of the investigations which are needed for scoring but are neither routinely performed nor requested by anaesthetists for low risk surgery were not available thus these patients had to be excluded.
ii. Not all patients had an HIV test and those with known HIV infection had an unknown viral load and immunological status which could have affected the interpretation of regression analysis on HIV status.

## Ethical approval

Ethical approval was obtained from Joint Research Ethics Committee for the University of Zimbabwe, College of Health Sciences and Parirenyatwa Group of Hospitals (JREC), JREC Ref: 290/14 and the Harare Central Hospital Ethics Committee Reference: HCHEC091014/67 and the Medical Research Council of Zimbabwe MRCZ ref: B767.

## Sources of funding

None. This research did not receive any specific grant from funding agencies in the public, commercial, or not-for-profit sectors.

## Author contribution

Allan Ngulube: project design, data collection, subject research, analysis and interpretation of data, writing and consent.

Edwin G. Muguti: subject research, analysis and interpretation of data, writing, editing.

Godfrey I. Muguti: project design, subject research, analysis and interpretation of data, writing, editing.

## Conflicts of interest

There is no conflict of interest.

## Trial registry number

UMIN Clinical Trials Registry - UMIN000034455.

https://upload.umin.ac.jp/cgi-open-bin/icdr_e/index.cgi.

## Guarantor

Allan Ngulube.

Godfrey I Muguti.

## Consent

A written informed consent was obtained from all patients who participated in the study.

## Provenance and peer review

Not commissioned externally peer reviewed.
