# Supplementary material for: Validation of POSSUM, P-POSSUM and the surgical risk scale in major general surgical operations in Harare: A prospective observational study
Source: Ann Med Surg (Lond). 2019 Mar 27;41:33–9. doi: 10.1016/j.amsu.2019.03.007 (PMC6475666; doi:10.1016/j.amsu.2019.03.007)
Supplement: Multimedia component 1 [file mmc1.docx]

| The STROCSS Guideline | | |
| --- | --- | --- |
| Item no. | **Item description** | Page Number |
| 1 | Title. The words “cohort” and the area of focus should appear in the title (e.g. disease, exposure/intervention or outcome). Whether the study is retrospective or prospective should also be stated. | 1  VALIDATION OF POSSUM, P-POSSUM AND THE SURGICAL RISK SCALE IN MAJOR GENERAL SURGICAL OPERATIONS IN HARARE: A PROSPECTIVE OBSERVATIONAL STUDY. This was a prospective observational cohort study. |
| 2a | Abstract - Introduction What is the background and scientific rationale for the research question. | 1  Raw mortality and morbidity, though commonly studied in surgical audit can nonetheless be misleading because of differences in preoperative and intraoperative findings of patients. There are some common scoring systems specifically designed to cater for case mix but these have not been tried locally. This study sought to validate these scoring systems and hopefully adopt them for our teaching hospitals. |
| 2b | Abstract - Methods - Describe the study design (cohort design, retrospective or prospective, single or multi-centre, etc), what was done to each group, how, when was it done and by whom. | 1  A prospective observational cohort study was conducted at two central hospitals in Harare  Two hundred and two patients undergoing a variety of major general surgical operations were recruited into the study. Results of physiological and intraoperative parameters collected from the patients’ records were scored according to POSSUM , P-POSSUM and SRS scores. Predicted mortality and morbidity rates of all these subjects were then compared to the observed rates. |
| 2c | Abstract - Results What was found. Give the results for the main outcomes. | 1  One hundred and eighty one patients participated (123 males, 58 females). Using the POSSUM morbidity score, the observed versus expected (O: E) ratio of 0.88 showed no difference ( p = 0.970). Using POSSUM , P-POSSUM and SRS mortality scores, O: E ratios of 0.74, 1.06 and 1.0 respectively were obtained, the differences were not significant ( p=0.650, p = 0.987 and 0.730). All three scores were comparable on the Receiver Operating Characteristic curve. The Physiological score independently predicted mortality (p<0.00001). |
| 2d | Abstract - Conclusion - What have we learned and what does it mean. Where should future research go. | 2  POSSUM, P-POSSUM and SRS scores are comparable and suitable for estimating outcomes after major surgery in Harare. A larger study inclusive of low risk patients is needed to generalise these findings across Zimbabwean patients. |
| 3 | Explain the scientific background and rationale for the cohort study. What are objectives, research questions and the hypotheses. | 4  The specific objectives of this study were to:   1. To determine if there is any significant difference between observed versus predicted operative mortality and morbidity scores in Harare using POSSUM, P-POSSUM and SRS. 2. To determine which perioperative risk factors have the greatest impact on mortality and morbidity |
| 4a | Registration and ethics State the research registry number in accordance with the declaration of Helsinki - "Every research study involving human subjects must be registered in a publicly accessible database before recruitment of the first subject" (this can be obtained from; ResearchRegistry.com or ClinicalTrials.gov or ISRCTN). Even retrospective studies should be registered prior to submission. | 12  The study was registered in UMIN Clinical Trials Registry - UMIN000034455 |
| 4b | Ethical Approval - State whether ethical approval was needed and if so, what the relevant judgement reference from the IRB or local ethics committee was? If ethical approval was not needed, state why. | 11  Permission to conduct the study was sought from the Medical Research Council of Zimbabwe ref MRCZ B/767, Joint Parirenyatwa Group of Hospitals and College of Health Sciences Research Ethics Committee JREC Ref:290/14, Ethics Committee at Harare Central Hospital and Clinical Directors of Parirenyatwa Group of Hospitals and Harare Central Hospitals. |
| 4c | Protocol - Was a research protocol developed apriori? Where can it be accessed. Was it published in a journal e.g. IJS Protocols, BMJ Open, etc, if so, provide the reference. | No: No protocol was published apriori |
| 5a | Study design - State the research is a cohort study and whether prospective or retrospective in design, whether single or multi-centre. | A prospective observational cohort study was done at 2 centres. |
| 5b | Setting - Describe the setting(s)and nature of the institution in which the patient was managed; academic, community or private practice setting? Location(s), and relevant dates, including periods of recruitment, exposure, follow-up, and data collection | 4  A prospective observational cohort study was done at Parirenyatwa Group of Hospitals (PGH) and Harare Central Hospital (HCH) over a 9 month period from January to September of 2015. All admitted general surgery patients aged 18 years and above undergoing at least a major surgical procedure were included and were followed up in the ward and outpatients department for a period up to 30 days post operation.  . |
| 5c | Cohort Groups - State the number of groups in the study. What interventions will each group receive? | 4  There was one major group all undergoing major surgery – the treatment will be standard, this group will be divided into two analysis groups, the observed outcomes and the other group being the expected outcome. |
| 5d | Sub-group – Analysis. Any planned sub-group analyses are specified / Describe any methods used to examine subgroups and interactions. | 4  There were 2 Major Subgroups- morbidity group and mortality group. Morbidity group - those with no morbidity and those with morbidity  Mortality group – those alive on Day 30 and those who die before day 30. Using a predesigned data collection tool, results from investigations done immediately preoperatively plus operative findings and post operative histology were collected and inputed. Mortality and morbidity risk was predicted by a computer program utilising the stated formulae (Equation 1-4). Complications were recorded as observed by the attending surgeons with confirmatory tests where necessary. Patients were followed up for a month in Outpatients Department (OPD) and a follow up phone call was done for those not available for review. The calculated risk scores for individual patients were stratified according to magnitude then compared with the actual observed number of mortalities or morbidities in each category with Chi-Square as a test for significance at 95% significance. Regression analysis of risk factors was also done with appropriate calculations for significance testing using statistical software. |
| 6a | Participants - State any eligibility (inclusion/exclusion) criteria and the sources and methods of selection of participants. Describe length and methods of follow-up. | 4  All admitted general surgery patients aged 18 years and above undergoing at least a major procedure, as defined by the British United Provident Association, were included. Patients were excluded if below the age of 18 years, if managed conservatively, if it was a day case or any procedure categorized as minor, or any case falling outside the scope of general surgery. Those also excluded were patients with more than 1 missing result or those requiring admission into a critical care unit post operatively but failed because of shortage of beds and those operated by surgical trainees with less than 2 years experience. |
| 6b | Recruitment - State the methods of how patients or participants were recruited to each group, over what time periods. | 4  All General Surgery patients aged 18years and above undergoing a minimum of a major procedure over a 9 month period from January 2015 to September 2015 were recruited. |
| 6c | Sample size calculation Whether there was calculation of margin of error or a prior analysis to determine study population, or mention of how appropriate study sample was determined. | 4  Sample size calculation was done using the Dobson’s formula.  Calculation:  n = z^2^  p (1-p)  d^2^  Description:  n= required sample size.  z= confidence level at 95% (standard value of 1.96)  p= estimated mortality after major surgery  d=margin of error at 5% (standard value of 0.05)  From the literature review a crude mortality rate of ( p) 12.3 % , inclusive of emergencies and elective cases . $n=\frac{1.96^{2}0.123(1-0.123)}{0.05^{2}}$   n = 165.8  The minimum sample size was therefore 166 patients. |
| 7a | Pre-intervention considerations - e.g. Patient optimisation: measures taken prior to surgery or other intervention e.g. treating hypothermia/hypovolaemia/hypotension in burns patients, ICU care for sepsis, dealing with anticoagulation/other medications and so on. | There was no deviation from standard treatment protocols and any patient who did not meet standard treatment protocols was excluded from the study. |
| 7b | Types of intervention(s) deployed - To include reasoning behind treatment offered (pharmacological, surgical, physiotherapy, psychological, preventive) and concurrent treatments (antibiotics, analgesia, anti-emetics, nil by mouth, VTE prophylaxis, etc). Medical devices should have manufacturer and model specifically mentioned. | None. No deviation from standard treatment protocols |
| 7c | Peri-intervention considerations - Administration of intervention (what, where, when and how was it done, including details for surgery; anaesthesia, patient position, use of tourniquet and other relevant equipment, preparation used, sutures, devices, surgical stage (1 or 2 stage, etc) and operative time. Pharmacological therapies should include formulation, dosage, strength, route and duration). Authors are encouraged to use figures, diagrams, photos, video and other multimedia to explain their intervention. | None. No deviation from standard treatment protocols. |
| 7d | Who performed the procedure(s) - Operator experience for each group (position on the learning curve for the technique if established, specialisation and prior relevant training). | 4  Surgery was perfomed by Consultants, Snr Registrars and Surgical Trainees with a minimum of 2 years training experience as guided by local protocols. |
| 7e | Quality control - What measures were taken to reduce inter or intra-operator variation. What measures were taken to ensure quality and consistency in the delivery of the intervention e.g. independent observers, lymph node counts, etc | 4  All the parameters are measurable and results were as reported by a standard laboratory. Definitions of outcomes were given before the study and were followed. |
| 7f | Post-intervention considerations - e.g. post-operative instructions and place of care. Important follow-up measures - diagnostic and other test results. Future surveillance requirements - e.g. imaging surveillance of endovascular aneurysm repair (EVAR) or clinical exam/ultrasound of regional lymph nodes for skin cancer. | None |
| 8 | Outcomes - What primary and secondary (if any) outcomes will be assessed and how are they defined. Definitions should be clear and precise. Appropriate references to validation of outcome measures used should be provided if they exist. | 4  Outcomes are Mortality and Morbidity. The observed outcomes were compared to expected outcomes with Chi Square test for significance. Comparison between the three risk prediction models was done with Receiver Operating Characteristic curve.  Definitions of morbidity by Copeland et al.  1. Hemorrhage: wound hemorrhage: local hematoma requiring evacuation. Deep hemorrhage: postoperative bleeding requiring re-exploration.  2. Chest infection: production of purulent sputum with positive bacteriological cultures, with or without chest radiography changes or pyrexia, or consolidation seen on chest radiograph.  3. Wound infection: wound cellulitis or the discharge of purulent exudates.  4. Urinary infection: the presence of >105 bacteria/ml with the presence of white cells in the urine, in previously clear urine.  5. Deep infection: the presence of an intraabdominal collection confirmed clinically or radiologically.  6. *Septicemia: positive blood culture.  7. Pyrexia of unknown origin: any temperature above 37 0C for more than 24 h occurring after the original pyrexia following surgery (if present) had settled, for which no obvious cause could be found.  8. Wound dehiscence: superficial or deep wound breakdown.  9. Deep venous thrombosis and pulmonary embolus: when suspected, confirmed radiologically by venography or ventilation/ perfusion scanning or diagnosed at post mortem.  10. Cardiac failure: symptoms or signs of left ventricular or congestive cardiac failure, which required an alteration from preoperative therapeutic measures.  11. Impaired renal function: arbitrarily defined as an increase in blood urea of >5mmol/l from preoperative levels.  12. *Hypotension: a fall in systolic blood pressure below 90 mmHg for more than 2 H as determined by sphygmomanometer or arterial pressure transducer measurement.  13. Respiratory failure: respiratory difficulty requiring emergency ventilation.  14. Anastomotic leak: discharge of bowel content via the drain, wound or abnormal orifice.  * In our study some patients were simply classed as septic shock implying septicaemia with hypotension requiring inotropic support |
| 9 | Statistical methods - Clearly outlined statistical tests used to compare the outcomes between an intervention group and a comparison group, state whether pre-existing differences and known confounders were controlled.  The statistical package used should be mentioned. | 5  The observed outcomes were compared to expected with chi-square as a test of significance and regression analysis of contributing factors. Statistical Package for Social Scientist (SPSS) version 16 and Epidemiological Information (EPI-Info 7) statistical software were used. |
| 10a | Participants recruited with a flow diagram - Report numbers involved in each group and use a flow diagram to show recruitment, non-participation, cross-over, withdrawal from the study with reasons. | 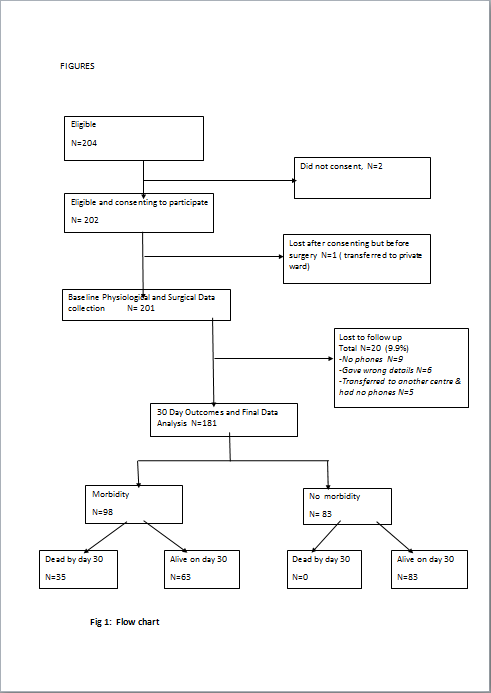 |
| 10b | Comparison between groups including a table - Provide a table comparing the demographic, clinical/prognostic features (co-morbidities, tumour staging, smoking status, etc) and relevant socioeconomic characteristics of each group and whether numerical differences are significant (using p-values and/or confidence intervals as appropriate). Were the groups matched and if so, how. | 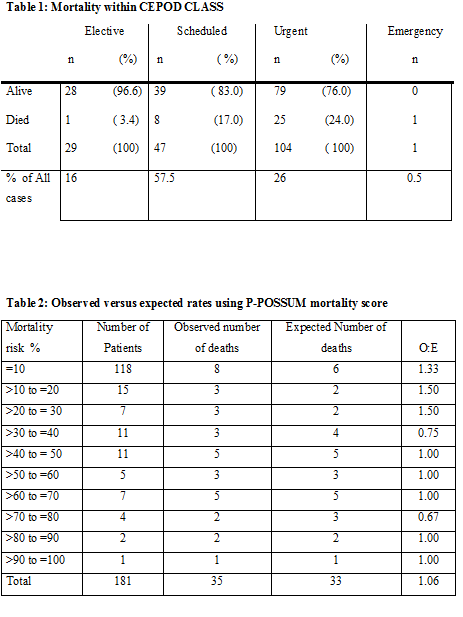 |
| 10c | Changes - Any changes in the interventions during the course of the study (how has it evolved, been altered or tinkered with, what learning occurred, etc) together with rationale and a diagram if appropriate. Degree of novelty for a surgical technique/device should be mentioned and a comment on learning curves should be made for new techniques/devices. | None |
| 11a | Outcomes and follow-up - Clinician assessed and patient-reported outcomes (when appropriate) should be stated for each group (size of effect with raw numbers and percentages) with inclusion of the time periods at which assessed. Relevant photographs/radiological images should be provided e.g. 12-month follow-up.Make it clear which confounders were adjusted for and which were not. | 6  Thirty five of 181 (19.3%) (17 PGH, 18 HCH) patients had died of surgery related problems after a 30 day follow up. The overall morbidity was 54%. Surgical experience was corrected for by excluding cases done by less experienced surgeons. Perioperative care was corrected for by excluding all cases that required critical care unit admission according to local treatment protocols but failed because of availability of beds. There was no correction of comorbid conditions like HIV and Diabetes. |
| 11b | Intervention adherence/compliance and tolerability - How was this assessed. Describe loss to follow-up (express as a percentage and a fraction) or cross-over between group and any explanations for them. | Fig1  There was a 10% loss to follow up , 20/201 patients. |
| 11c | Complications and adverse or unanticipated events - Described in detail and ideally categorised in accordance with the Clavien-Dindo Classification. How they were prevented, mitigated, diagnosed and managed. Blood loss, wound complications, re-exploration/revision surgery, 30-day post-op and long-term morbidity/mortality may need to be specified. | Complications were as defined by the original authors of the POSSUM score. This study was comparing how the score perfoms and therefore there was no deviation from standard treatment protocols. |
| 12 | Summarise key results | 1  Using the POSSUM morbidity score, the observed versus expected (O: E) ratio of 0.88 showed no difference ( p = 0.970). Using POSSUM , P-POSSUM and SRS mortality scores, O: E ratios of 0.74, 1.06 and 1.0 respectively were obtained, the differences were not significant ( p=0.650, p = 0.987 and 0.730). All three scores were comparable on the Receiver Operating Characteristic curve. The Physiological score independently predicted mortality (p<0.00001). |
| 13 | Discussion of the relevance of the findings and rationale for conclusions - Relevant literature, implications for clinical practice guidelines, how have the indications for a new technique/device been refined and how do outcomes compare with established therapies and the prevailing gold standard should one exist and any relevant hypothesis generation. The rationale for any conclusions. | 7-10  From our study we found that the level and experience of clinicians did not seem to have an impact on mortality and morbidity with no significant difference in observed versus expected mortality (p= 0.872). We therefore postulate that surgical trainees may possibly use these scoring systems for longitudinal assessment of their own performance. The area under the curve for POSSUM morbidity score is 0.775 ( p < 0.0001) agreeing with existing literature that shows that the score has good discrimination for picking those who will get a morbidity and also agreeing with literature that POSSUM scores are among the most useful for general surgery.  Both the physiological score and operative scores correlated significantly with morbidity with p-values 0.002 and 0.007 and also with mortality with p-values <0.00001 and 0.0036 respectively. We therefore believe that the physiological POSSUM score can be used for preoperative counselling of patients and allocation of resources.  The area under the ROC curves for POSSUM, SRS and P-POSSUM showed no statistically significant difference and all were close to 80% of the area showing that all the three scores have good discrimination for picking those who will become a mortality. |
| 14 | Strengths and limitations of the study | 11  i.)The study had no funding and as a result some of the investigations which are needed for scoring but are neither routinely performed nor requested by anaesthetists for low risk surgery were not available thus these patients had to be excluded.  ii.) Not all patients had an HIV test and those with known HIV infection had an unkown viral load and immunological status which could have affected the interpretation of regression analysis on HIV status. |
| 15 | State what needs to be done next, further research with what study design(s). | 10  If funding is available a much larger similar study including the lower risk stratas would need to be done. |
| 16 | State the key conclusions from the study and key directions for future research | 2  POSSUM, P-POSSUM and SRS scores are comparable and suitable for estimating outcomes after major surgery in Harare. A larger study inclusive of low risk patients is needed to generalise these findings across Zimbabwean patients. |
| 17a | State any conflicts of interest | None |
| 17b | State any sources of funding | None |
